# BH3 mimetic ABT-263 enhances the anticancer effects of apigenin in tumor cells with activating EGFR mutation

**DOI:** 10.1186/s13578-019-0322-y

**Published:** 2019-07-23

**Authors:** Yihong Zhan, Yue Wang, Miao Qi, Panpan Liang, Yu Ma, Ting Li, Hui Li, Congmei Dai, Zhifeng An, Yitao Qi, Hongmei Wu, Huanjie Shao

**Affiliations:** National Engineering Laboratory for Resource Developing of Endangered Chinese Crude Drugs in Northwest of China, Key Laboratory of the Ministry of Education for Medicinal Resources and Natural Pharmaceutical Chemistry, College of Life Sciences, Shaanxi Normal University, Xi’an, 710119 Shaanxi China

**Keywords:** Apigenin, EGFR, ABT-263, FoxO3a, Noxa

## Abstract

**Background:**

Mutated epidermal growth factor receptor (EGFR) is one of the most successful targets in cancer targeted therapy. While this treatment has benefited many patients with an activating EGFR mutation (EGFRm), almost all those who initially benefited will eventually develop acquired drug resistance (ADR) after a certain period of time. New therapeutic strategies need to be explored to treat EGFRm tumors and overcome or minimize this recurring ADR.

**Results:**

Our data showed that apigenin alone has only mild inhibitory effects on EGFRm tumor cells. By drug screening, we found that ABT-263 can significantly enhance the antitumor activities of apigenin in tumor cells harbouring an activating EGFR mutation and AZD9291-resistant H1975 cells. Mechanistically, apigenin upregulated the expression of Noxa in EGFRm tumor cells by targeting the AKT-FoxO3a pathway, thereby synergizing with ABT-263 to suppress tumor cell growth and proliferation in vitro and in vivo.

**Conclusions:**

Our study provides a rationale for the clinical application of the combination treatment of apigenin and BH3 mimetics in the treatment of EGFRm tumors.

**Electronic supplementary material:**

The online version of this article (10.1186/s13578-019-0322-y) contains supplementary material, which is available to authorized users.

## Background

Cancer is one of the common causes of human death worldwide. The development of cancer is closely related to the mutation and abnormal expression of proto-oncogenes such as epidermal growth factor receptor (EGFR) [1]. According to the statistical analysis of the Catalogue of Somatic Mutation In Cancer (COSMIC) database, the abnormal expression or mutation of EGFR gene exists in almost all solid tumors, and the proportion of EGFR gene mutation in non-small cell lung cancer (NSCLC) patients are up to 26% or more.

EGFR is a member of the tyrosine kinase type I receptor subfamily and is widely distributed on the outer membrane of epithelial cells. It is one of the most studied receptor tyrosine kinases (RTKs) owing to its general role in signal transduction and oncogenesis. EGFR is activated when it binds to an extracellular ligand. Activated EGFR transmits the external signal stimulus to activate its downstream signaling pathways, such as the mitogen-activated protein kinase/extracellular signal-regulated kinase (MAPK/ERK), phosphoinositide 3-kinase (PI3K)-AKT and STAT3 pathways, in turn regulating cell growth, proliferation, differentiation and apoptosis [2–4]. Therefore, abnormal expression or an activating mutation of the EGFR gene will irreversibly lead to cancer [5, 6].

An activating EGFR mutation (EGFRm) is one of the main driver causes of oncogenesis for various cancers, including NSCLC. Thus, EGFR is an ideal target for cancer therapy. In recent years, tumor immunotherapy has provided a promising strategy for the cure of various cancers. Unfortunately, reports have shown that EGFRm tumors are not sensitive to treatment with PD1/PD-L1 inhibitors. EGFRm patients have weaker benefits from receiving PD1/PD-L1 inhibitors and may not be suitable for PD1/PD-L1 inhibitor treatment [7–10]. Currently, EGFRm tumors are widely treated with receptor tyrosine kinase inhibitors (TKIs). Additionally, the therapeutic strategy has achieved remarkable results in clinical applications. For example, in the treatment of NSCLC, the first generation of EGFRm inhibitors, gefitinib (Iressa) and erlotinib (Troquet, Tarceva), has been approved by the FDA and has achieved great clinical success [11, 12]. However, clinically, when EGFRm patients are treated with those first- or second-generation EGFR TKIs, almost all those who initially benefited will eventually develop acquired drug resistance (ADR) after a certain period of time [13–15]. Studies have shown that one of the root causes of tumor resistance is the occurrence of a second site mutation in the EGFR gene. Among them, EGFR-T790M mutation accounts for more than 50% of clinically resistant patients [16, 17]. Osimertinib (AZD9291), a third-generation EGFR TKI, was developed for the treatment of metastatic *EGFR* T790M mutation-positive NSCLC. However, resistance to AZD9291 has been reported, and *EGFR* C797S mutation was regarded as a major mechanism for resistance to T790M-targeting EGFR inhibitors [18–20]. To treat EGFRm tumors and overcome or minimize this recurring ADR, we need to explore novel therapeutic strategies to block the EGFR signaling pathway and treat tumors containing EGFRm.

Apigenin is a natural flavonoid found in our daily consumption of fruits and vegetables and Chinese herbal medicines. Studies have shown that apigenin serves multiple physiological functions, such as strong anti-inflammatory, antioxidant, antibacterial and antiviral activities and blood pressure reduction [21–24]. Recently, apigenin has been widely investigated for its anticancer activities. Our and other researchers’ work have shown that apigenin can target multiple singling pathways, including the MAPK, AKT, STAT and NF-κB pathways, to trigger tumor cell apoptosis and induce cell cycle arrest [25, 26]. Given that MAPK, AKT and STAT3 are major downstream signaling pathways of EGFR, apigenin seems to be a potential drug candidate to treat EGFRm tumors.

In the present study, we found that apigenin alone mildly suppressed the growth and proliferation of tumor cells with activating EGFR mutations, while ABT-263 can significantly enhance the antitumor effects of apigenin on EGFRm tumor cells. Mechanistic analysis showed that apigenin upregulated Noxa expression by inhibiting the AKT-FoxO3a signaling pathway to synergize with ABT-263 to suppress the growth and proliferation of EGFRm tumor cells in vitro and in vivo.

## Results

### Apigenin mildly inhibits proliferation of tumor cells with an activating EGFR mutation

**Fig. 1:**
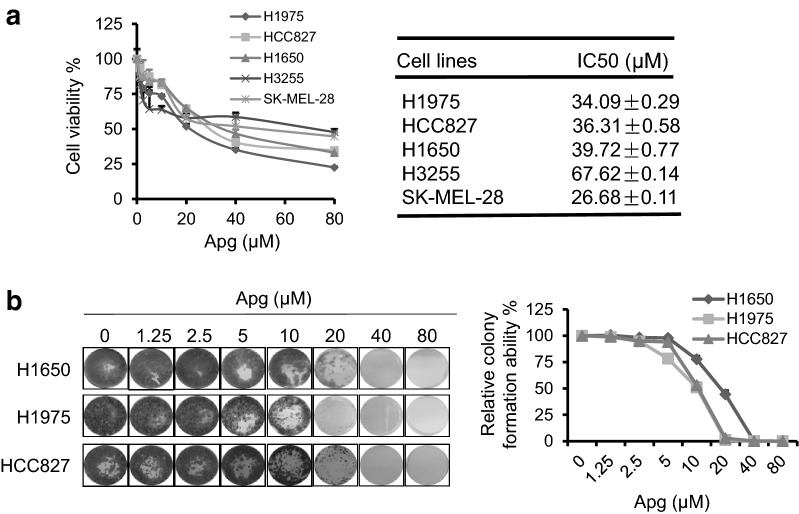
Apigenin has mild antitumor effects on cancer cells with activating EGFR mutation. **a** H1975, HCC827, H1650, H3255 and SK-MEL-28 cells were treated with increasing doses of apigenin (Apg) for 2 days. Cell viability was assessed using MTT assay. IC50 was analyzed by GraphPad Prism 7.00. **b** Cells were seeded in 24-well plates and treated with increasing doses of Apg as indicated for 14 days. The adherent cells were then stained with crystal violet for the colony formation assay (left). Colony formation ability was analyzed (right)

As reported previously, apigenin can target multiple downstream signaling pathways of EGFR and has a broad antitumor effect [25, 26]. To study the inhibitory effects of apigenin on EGFRm tumor cells, we treated a series of tumor cells harboring EGFRm with dosed apigenin for 2 days. As shown in Fig. 1a, consistent with a previous report, apigenin showed a concentration gradient inhibition of tumor cell growth and proliferation. Additionally, colony formation experiments confirmed that apigenin alone has significant dose-dependent inhibitory effects on the clonal formation of EGFRm tumor cells (Fig. 1b). Although apigenin has potential antitumor effects on those EGFRm cells, we noticed that apigenin needs a very high dosage to inhibit EGFRm tumor cell growth and proliferation. The IC50 value of apigenin in those tumor cells far exceed the maximum concentration that apigenin can achieve in the body [27, 28]. Therefore, although apigenin can effectively inhibit EGFRm tumor cells, it often requires a higher concentration to achieve better inhibition in those tumor cells.

### ABT-263 significantly enhances the anticancer effects of apigenin in tumor cells containing activating EGFR mutations

To enhance the anticancer efficacy of apigenin in EGFRm tumor cells, we explored a series of traditional Chinese medicine monomers and small-molecule targeted inhibitors to identify a candidate that could synergistically interact with apigenin to suppress EGFRm tumor cells. We exploited H1975 cells as an in vitro cell model to analyze the antitumor effects of the co-treatment and found that BH3 mimetic of ABT-263 could significantly enhance the antitumor effect of apigenin to suppress tumor cell growth and proliferation (Additional file 1: Figure S1).

**Fig. 2:**
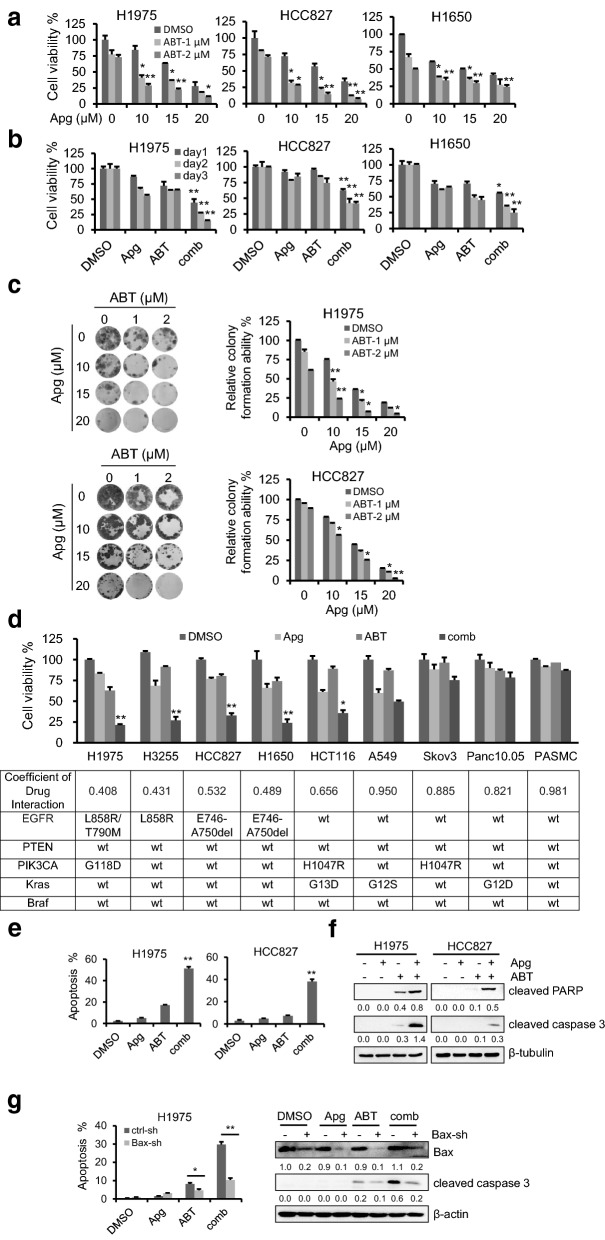
ABT-263 significantly enhances the anticancer effects apigenin in tumor cells containing activating EGFR mutation. H1975, HCC827 and H1650 cells were incubated with the indicated dosages of apigenin (Apg) or ABT-263 (ABT), alone or the combination (comb) for 1 day (**a**), or were treated with Apg (15 µM), ABT (2 µM) or their combination for up to 3 days (**b**), and the cell viability were detected by MTT assay. **c** H1975 and HCC827 cells were seeded in 24-well plates and treated with the indicated dosages of Apg or ABT, alone or the comb for 14 days. The adherent cells were then stained with crystal violet for the colony formation assay. **d** Tumor cells with different genetic background and immortalized human Pulmonary Artery Smooth Muscle cells were treated with the indicated drugs for 1 day. The cell viability rates were examined by MTT and the Coefficient of Drug Interaction (CDI) values were calculated. **e**, **f** Cells were treated with Apg (15 µM) and ABT (2 µM), alone or in combination for 1 day. Apoptotic cell death was analyzed (**e**), and cleaved PARP and cleaved caspase 3 were examined by Western blotting. β-Tubulin was detected as an endogenous loading control (**f**). Data represent mean ± SD (n = 3). **p < 0.01 vs single compound treatment group. **g** Bax was knocked down by shRNA in H1975 and HCC827 cells, and the cells were treated with drugs as described in **e**, followed by cell apoptosis analysis (left) and Western blotting analysis of Bax and cleaved caspase 3 (right). The data represents mean ± SD (n = 3). *p < 0.05, and **p < 0.01 vs ctrl-sh group

To validate the roles of ABT-263 in enhancing apigenin against EGFRm tumor cells, H1975, HCC827 and H1650 cells were employed. As shown in Fig. 2a and b, the combination of apigenin and ABT-263 showed dose- and time-dependent synergistic antitumor effects than each compound alone. Additionally, the colony formation assay confirmed that ABT-263 significantly enhanced the effect of apigenin on EGFRm tumor cells (Fig. 2c). Furthermore, the anti-tumour efficacy of co-treatment of apigenin and ABT-263 in EGFRm cells was examined using the Operetta High Content Imaging System. As shown in Additional file 1: Figure S2, while minimal cell growth inhibition was detected when the cells were treated with apigenin or ABT-263 alone, there was a significant decrease in cell proliferation when the cells were co-treated with apigenin and ABT-263.

As reported previously, apigenin can synergize with various compounds to exert antitumor effects on various tumors [29, 30]. To test whether the combination of apigenin and ABT-263 can selectively suppress tumor cells with EGFRm, we analyzed a series of tumor cells with various gene mutations and immortalized human Pulmonary Artery Smooth Muscle Cells (PASMC) with apigenin and ABT-263 alone or in combination. As shown in Fig. 2d, the coadministration of two drugs has a significant synergistic inhibitory effect on EGFRm tumor cells but less cytotoxicity on cells with normal EGFR or little toxic side effects on immortalized epithelial cells.

Considering that the Operetta High Content Imaging System analysis shows that the combination caused serious tumor cell death, we tested cell death annexin-V and PI staining using flow cytometry analysis. We found that the cotreatment of apigenin and ABT-263 significantly triggered cell apoptosis in EGFRm cells (Fig. 2e). Western blotting analysis also showed that the combination treatment of apigenin and ABT-263 induced greater cleavage of caspase 3 and PARP than the drugs did alone, further demonstrating a synergistic interaction between ABT-263 and apigenin in EGFRm tumor cells (Fig. 2f). Finally, the occurrence of endogenous apoptosis was closely related to the activation of Bax on mitochondria. As shown in Fig. 2g, downregulation of Bax by shRNA significantly rescued cells from cotreatment-induced cell apoptosis, indicating that the combination treatment induces endogenous apoptosis in EGFRm tumor cells.

### ABT-263 synergizes with apigenin to suppress engineered BaF3 cells harbouring EGFR mutations

**Fig. 3:**
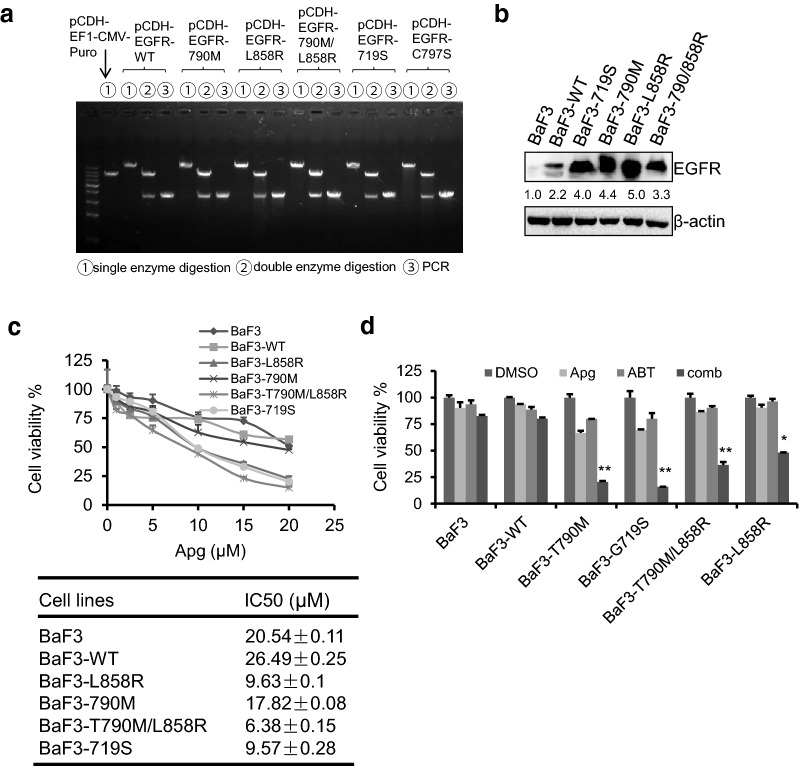
ABT-263 synergized with apigenin to suppress engineered BaF3 cells harboring EGFR mutation. **a** Agarose gel electropherogram of plasmid of pCDH-EGFR-WT, -T790M, -L858R, -T790M/L858R, -G719S and -C797S digestion and PCR of EGFR gene cDNA ORF clone sequences. **b** Engineered BaF3 cells with or without EGFRm were harvested and EGFR protein levels were detected by Western blotting. **c** Cells were treated with increasing doses of apigenin for 2 days. Cell viability rates were examined using MTT assay (upper) and the IC50 value was calculated (lower). **d** Cells were seeded in 24-well plates and treated with Apg (5 µM) and ABT (0.5 µM), alone or comb for 1 day. Cell viability rates were analyzed using MTT assay. Data represent mean ± SD (n = 3). *p < 0.05, and **p < 0.01 vs single compound treatment group

To further investigate the specific inhibition on EGFRm cells by the cotreatment of apigenin and ABT-263, we constructed EGFR expression plasmids, including EGFR-wt, -719S, -T790M, -L858R -T790M/L858R and -C797S, and introduced them into BaF3 cells to construct BaF3-engineered cell lines harboring the EGFRm plasmid (Fig. 3a, b). It is worth noting that we failed to get the BaF3-EGFR-C797S engineering cells for reasons we don’t know so far. Therefore, the antitumor effect of the combination was carried out in other engineering cells. As shown in Fig. 3c, apigenin alone showed only limited cytotoxicity on BaF3 and BaF3-EGFR-wt (IC50 > 20 µM). Interestingly, BaF3 cells containing EGFR mutations were more sensitive to apigenin treatment, showing an IC50 value of approximately 10 µM. Furthermore, we used these cells to examine the cytotoxicity of the cotreatment of apigenin and ABT-263. The results showed that the combination has enhanced antitumor effects on BaF3-engineered cells containing exogenous mutated EGFR, while the BaF3 parental cells and BaF3-EGFR-wt cells were resistant to the cotreatment of apigenin and ABT-263 (Fig. 3d).

### ABT-263 and apigenin significantly inhibit the growth and proliferation of AZD9291-resistant H1975 cells

**Fig. 4:**
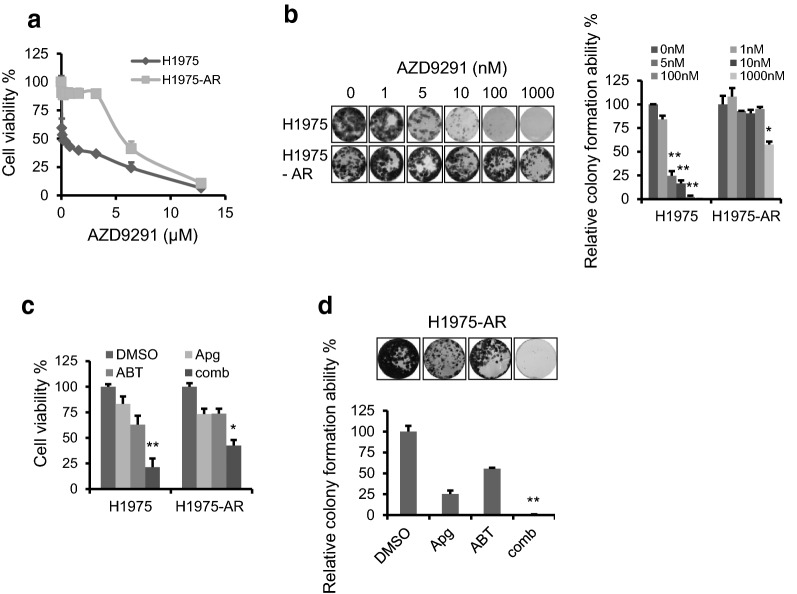
ABT-263 and apigenin significantly suppressed the growth and proliferation of AZD9291-resistant H1975 cells. **a** H1975 parental and H1975 AZD9291-resistant (H1975-AR) cells were treated with increasing doses of AZD9291 for 2 days. Cell viability was assessed using MTT assay. **b** H1975 and H1975-AR cells were seeded in 24-well plates and treated with increasing doses of AZD9291 for 21 days. The adherent cells were then stained with crystal violet for the colony formation assay (left) and colony formation ability was analyzed (right). **c** H1975 and H1975-AR cells were seeded in 24-well plates and were treated with Apg (15 µM) and ABT (2 µM), alone or comb for 1 days, cell viability rates were examined by MTT. **d** H1975-AR cells were seeded in 24-well plates and treated with Apg (15 µM) and ABT (2 µM), alone or comb for 14 days for the colony formation assay (upper), colony formation ability was analyzed (lower). The data represents mean ± SD (n = 3). *p < 0.05, and **p < 0.01

Clinically, a challenge for EGFRm tumor treatment with TKIs is the frequent ADR in tumor cells, leading to tumor treatment failure or tumor recurrence. To test whether the combination of ABT-263 and apigenin also plays a role in the cells resistant to the third-generation EGFR inhibitor AZD9291, we first constructed a model of the AZD9291-resistant H1975 cell line (H1975-AR). H1975 cells were cultured and chronically exposed to escalating doses of AZD9291 for approximately ten months in vitro. As shown in Fig. 4a, H1975-AR cells were significantly more tolerant to AZD9291 treatment than H1975 parental cells. Additionally, the IC50 value was increased from 11 nM (parental cells) to approximately 6.2 µM (H1975-AR cells). Meanwhile, the colony formation assay confirmed that H1975-AR cells were more resistant to AZD9291 (Fig. 4b). Sequencing analysis of H1975-AR cells revealed that no new site mutations occurred in EGFR in H1975-AR cells (Additional file 1: Figure S3). The combination of apigenin and ABT-263 was administered to treat H1975 and H1975-AR cells. As shown in Fig. 4c and d, the cotreatment of apigenin and ABT-263 also showed good inhibitory effects on H1975-AR cells.

### Noxa plays a key role in the cotreatment of apigenin and ABT-263-induced apoptosis

**Fig. 5:**
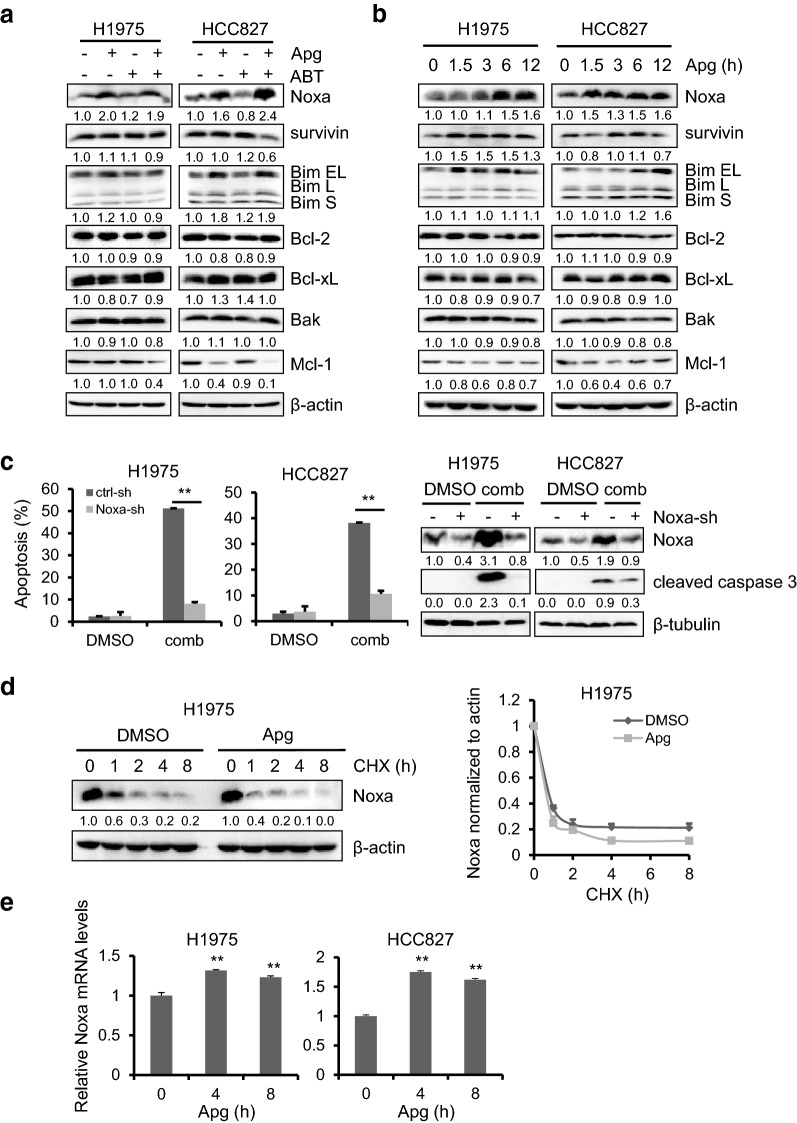
Noxa modulation by apigenin contribute to coadministration-induced cell killing. **a** H1975 and HCC827 cells were treated with Apg (15 µM), ABT (2 µM) alone or comb for 1 day. Cells were harvested and Bcl-2 family members were detected by Western blotting. **b** H1975 and HCC827 cells were treated with Apg (15 µM) for up to 12 h, Bcl-2 family members were examined by Western blotting. **c** H1975 and HCC827 cells infected with the sh-Noxa lentivirus were treated with drugs as described in **a**. Apoptotic cell death was analyzed (left), and expression of Noxa and cleaved caspase 3 were analyzed by Western blotting (right). **d** H1975 and HCC827 cells were treated with 10 µM CHX alone or in combination with 15 µM Apg for up to 8 h. Noxa expression was examined by Western blotting (left), and its quantification (right) was examined by ImageJ (the bands densitometry of Noxa were measured by ImageJ and normalized to β-actin, then compared to DMSO-treated control). **e** H1975 and HCC827 cells were treated with Apg (15 µM) for up to 8 h. The total RNA was extracted and the Noxa mRNA level was quantified by real-time PCR. The data represents mean ± SD (n = 3). **p < 0.01

To determine the mechanisms underlying the induction of cell death by the cotreatment of apigenin and ABT-263, we analyzed the expression of Bcl-2 family members. As shown in Fig. 5a, among all Bcl-2 family proteins tested, Noxa was significantly upregulated upon apigenin treatment, alone or in combination. Furthermore, we found that Noxa was time dependently upregulated by apigenin in H1975 and HCC827 cells (Fig. 5b). To verify whether the upregulation of Noxa by apigenin played a key role in the induction of tumor cell apoptosis, Noxa was knocked down by shRNA in H1975 and HCC827 cells. As expected, downregulation of Noxa significantly rescued cells from cotreatment-induced cell apoptosis, shown as a reduced apoptotic cell number and decreased cleaved caspase 3 expression (Fig. 5c).

We further explored how apigenin regulates the expression of Noxa. H1975 cells were treated with cycloheximide in the presence or absence of apigenin for up to 8 h. Cells were harvested for Western blotting analysis. As shown in Fig. 5d, the presence of apigenin did not contribute to Noxa downregulation by cycloheximide, suggesting that the stimulation of Noxa expression by apigenin might occur at the transcriptional level. Next, we examined Noxa mRNA using real-time PCR. After 4 and 8 h of treatment with apigenin, Noxa mRNA was increased by more than 30% in apigenin-treated H1975 cells at both 4 and 8 h compared with vehicle-treated control. Similar results were observed in HCC827 cells as well.

### AKT and ERK signaling pathways contribute to the synergistic interaction between apigenin and ABT-263

**Fig. 6:**
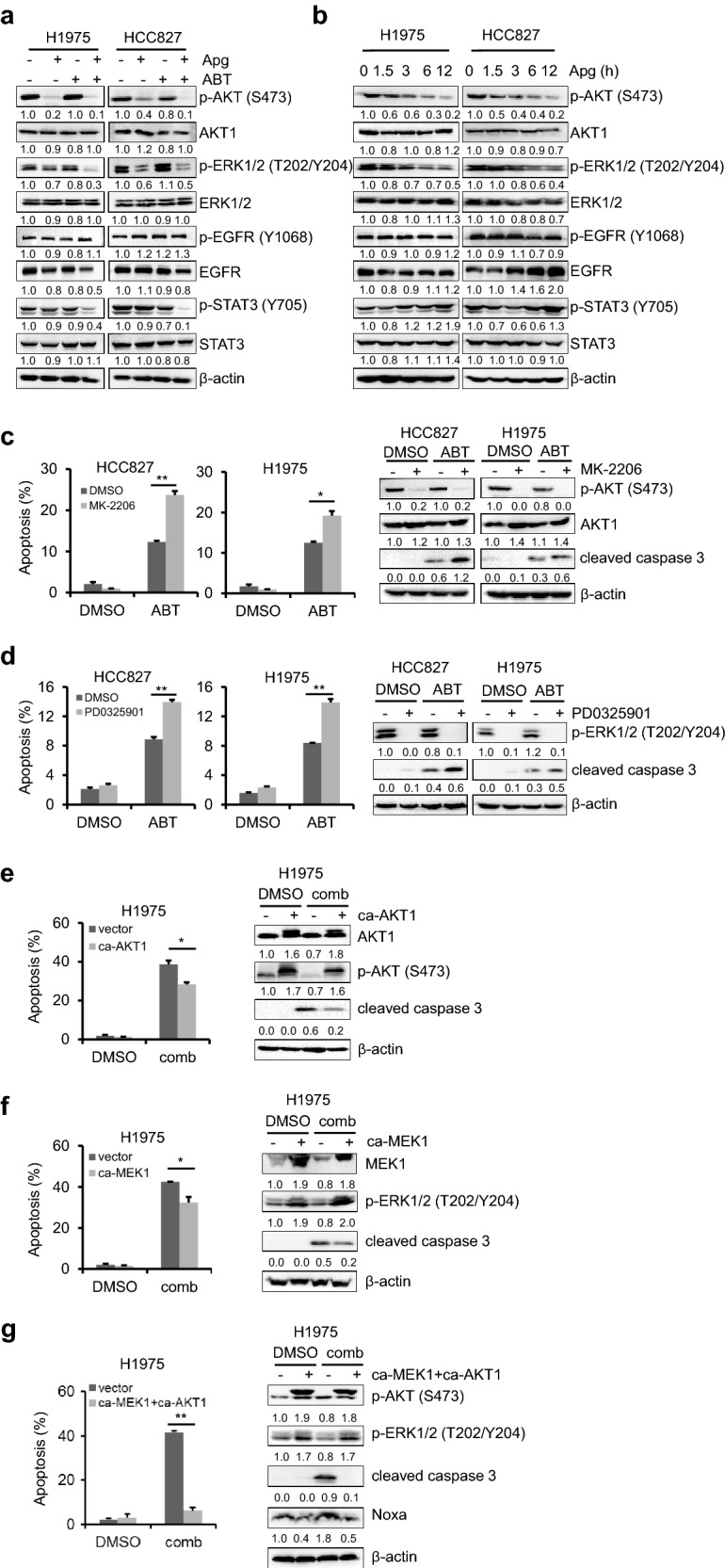
AKT and ERK signaling pathways contributed to synergistic interaction between apigenin and ABT-263. **a** H1975 and HCC827 cells were treated with Apg (15 µM), ABT (2 µM) alone or in combination for 1 day. Cells were harvested, and the major downstream pathway molecules of EGFR were examined by Western blotting. **b** H1975 and HCC827 cells were treated with 15 µM Apg for up to 12 h, the total and phosphorylated AKT, ERK1/2, STAT3 and EGFR were analyzed by the immunoblotting. **c** H1975 and HCC827 cells were treated with MK-2206 (1 µM) and ABT (2 µM), alone or in combination for 1 day. The apoptotic cell death were analyzed (left), the total and phosphorylated-AKT and cleaved caspase 3 were detected by Western blotting (right). **d** H1975 and HCC827 cells were treated with PD0325901 (2 µM) and ABT (2 µM), alone or in combination for 1 day. The apoptotic death rates were analyzed (left), and the total and phosphorylated-ERK1/2 and cleaved caspase 3 were detected by Western blotting (right). **e** H1975 cells transfected with ca-AKT1 or empty vector were treated with DMSO or the combination of Apg and ABT for 24 h. The apoptotic death rates were analyzed (left), AKT1 and cleaved caspase 3 were determined by Western blotting (right). **f** H1975 cells transfected with ca-MEK1 or empty vector were treated with DMSO or the combination of Apg and ABT for 24 h. The apoptotic death rates were analyzed (left), MEK1 and cleaved caspase 3 were detected by Western blotting (right). **g** H1975 cells cotransfected with ca-MEK1and ca-AKT1 or empty vector were treated with DMSO or the combination of Apg and ABT for 24 h. The apoptotic death rates were analyzed (left), and p-AKT, p-ERK, cleaved caspase 3 and Noxa were detected by Western blotting (right). The data represents mean ± SD (n = 3). *p < 0.05

To explore the mechanism by which apigenin regulated Noxa expression and synergized with ABT-263 to inhibit EGFRm tumor cells, we analyzed the phosphorylation status of EGFR and its downstream signalling pathways, including MAPK, PI3K-AKT and STAT3. The results showed that the phosphorylation of AKT and ERK1/2 was significantly suppressed by apigenin treatment alone or in combination with ABT-263 (Fig. 6a). Furthermore, we found that the activation of AKT and ERK was inhibited by apigenin in a time-dependent manner (Fig. 6b).

To determine the role of AKT and ERK inactivation in the apigenin and ABT-263 synergistic interaction, we inhibited AKT or ERK with the selective small-molecule inhibitor MK-2206 or PD0325901, respectively, to mimic the action of apigenin in H1975 and HCC827 cells. As shown in Fig. 6c and d, inhibition of either AKT or ERK enhanced ABT-263-triggered cell death dramatically in both cell lines. Additionally, enhanced cleavage of caspase 3 was also observed in the presence of MK-2206 or PD0325901 (Fig. 6c, d). Furthermore, exogenously overexpressing either constitutively activated AKT1 (ca-AKT1) or constitutively active form of MEK1 (ca-MEK1) only partially rescued EGFRm cell apoptosis induced by apigenin and ABT-263 (Fig. 6e, f). In addition, exogenously overexpression of both the ca-AKT1 and ca-MEK1 could significantly protect H1975 cells from the cotreatment of apigenin and ABT-263-induced cytotoxicity (Fig. 6g). And the upregulation of Noxa induced by combination treatment of apigenin and ABT-263 was reverse by the ectopic expression of ca-AKT1 and ca-MEK1 (Fig. 6g). Together, those results suggested that inactivation of AKT and ERK by apigenin contributes to coadministration of ABT-263 and apigenin-induced antitumor responses in EGFRm tumor cells.

### Dephosphorylation of FoxO3a by apigenin upregulates Noxa expression in EGFRm tumor cells

**Fig. 7:**
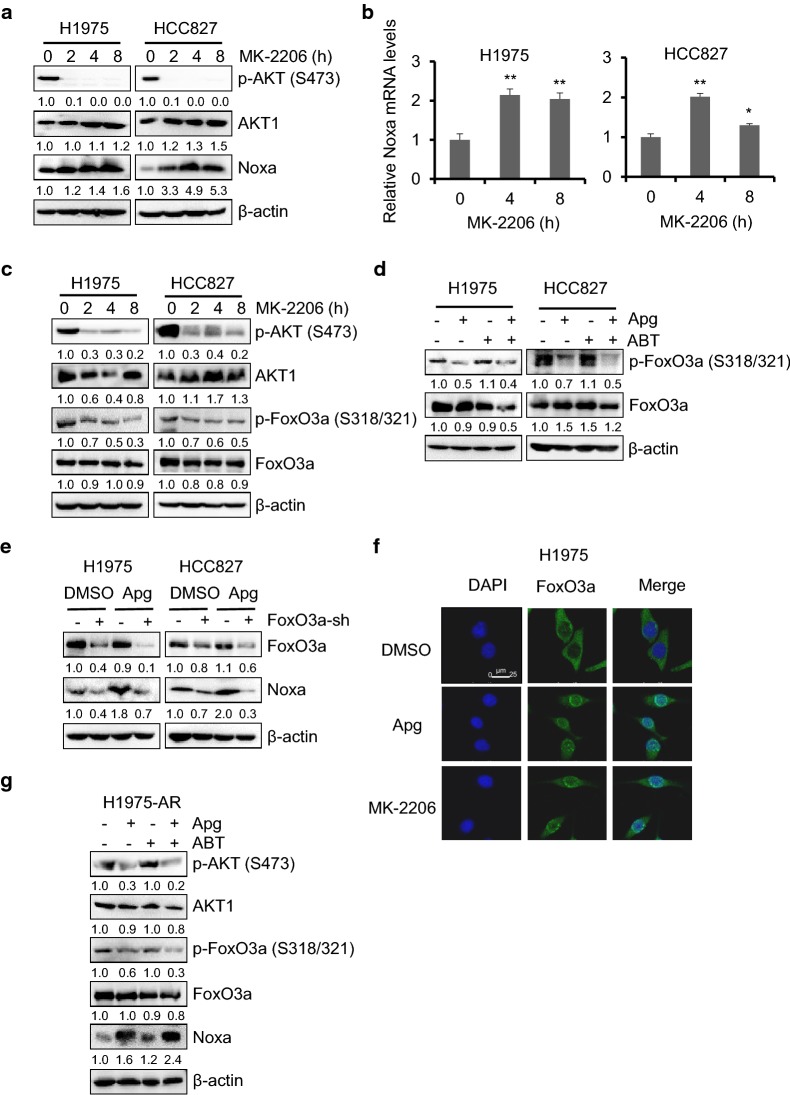
Dephosphorylation of FoxO3a by apigenin upregulated Noxa expression in EGFRm tumor cells. **a** H1975 and HCC827 cells were treated with MK-2206 (1 µM) for up to 8 h. Cells were harvested and the expression levels of AKT and Noxa were detected by the immunoblotting. **b** H1975 and HCC827 cells were treated with 1 µM MK-2206 for up to 8 h. Then the total RNA was extracted and the Noxa mRNA level was quantified by real-time PCR. The data represents mean ± SD (n = 3). *p < 0.05, and **p < 0.01. **c** H1975 and HCC827 cells were treated as described in **a**, total and phosphorylated AKT and FoxO3a was examined by Western blotting. **d** H1975 and HCC827 cells were treated with Apg (15 µM), ABT (2 µM) alone or comb for 1 day. Cells were harvested, and the expression levels of FoxO3a were detected by the immunoblotting. **e** H1975 and HCC827 cells infected with the ctrl-sh or FoxO3a-sh lentivirus were treated with 15 µM Apg. Apoptotic death rates were analyzed, and expression of FoxO3a and Noxa were analyzed by Western blotting. **f** Immunofluorescence staining of FoxO3a in H1975 cells. Cells were treated with apigenin or MK-2206 for 8 h. Cells were then stained for FoxO3a and nucleus using Alexa Fluor 488-conjugated antibody (green) and DAPI (blue), respectively. Representative staining cells are shown. **g** H1975-AR cells were treated with Apg (15 µM) and ABT (2 µM), alone or in combination for 1 day. Cells were harvested, and the expression levels of p-AKT, AKT, p-FoxO3a, FoxO3a and Noxa were examined by Western blotting

To investigate whether the upregulation of Noxa by apigenin is associated with the inactivation of AKT or ERK, we treated H1975 and HCC827 cells with PD0325901 or MK-2206, respectively, for up to 8 h, and the changes in Noxa expression was detected by Western blot analysis. As shown in Fig. 7a, AKT inactivation by MK-2206 significantly caused Noxa upregulation in a time-dependent manner, while ERK inhibition by PD0325901 did not upregulate Noxa expression (Additional file 1: Figure S4), indicating that AKT inactivation may play a key role in Noxa upregulation by apigenin. Furthermore, H1975 and HCC827 cells were treated with MK-2206 for up to 8 h, and Noxa mRNA was examined by real-time PCR. As expected, Noxa mRNA was increased significantly in H1975 cells at both 4 and 8 h compared with DMSO-treated control (Fig. 7b). Similar results were also observed in HCC827 cells (Fig. 7b).

Forkhead box O3, also known as FoxO3 or FoxO3a, is a downstream target of AKT. Phosphorylation of FoxO3a by AKT sequestrates FoxO3a in the cytoplasm, thus blocking its function as a transcription factor and contributing to cell survival, growth and proliferation [31, 32]. As reported previously, Noxa could be regulated by FoxO3a at the transcriptional level [33, 34]. First, we found that MK-2206 inhibited AKT activation and dephosphorylated FoxO3a at the sites of S318/S321 in a time-dependent manner in H1975 and HCC827 cells (Fig. 7c). Additionally, the phosphorylation of FoxO3a was suppressed by apigenin treatment alone or in combination with ABT-263 in H1975 and HCC827 cells (Fig. 7d). To examine whether FoxO3a is involved in apigenin-induced Noxa upregulation, we knocked down FoxO3a expression using shRNA. Knockdown of endogenous FoxO3a abrogated the apigenin-induced increase in the Noxa level in H1975 and HCC827 cells (Fig. 7e), indicating that the AKT-FoxO3a pathway mediates apigenin-induced Noxa upregulation in EGFRm tumor cells. To figure out how FoxO3a mediated Noxa upregulation upon apigenin treatment, we examined the subcellular localization of FoxO3a in H1975 cells in the presence or absence of apigenin. As shown in Fig. 7f, immunofluorescence staining results demonstrated that FoxO3a was accumulated in the nucleus upon apigenin treatment in H1975 cells. Similar results were also observed when those EGFRm cells were treated with MK-2206. Interestingly, the decreased phosphorylation of AKT and FoxO3a, and upregulation of Noxa were observed in H1975-AR cells treated with apigenin alone or the combination of apigenin and ABT-263 (Fig. 7g), indicating that apigenin may also target those molecules in H1975-AR cells. Together, those results suggest that the dephosphorylation of FoxO3a plays an important role in mediating apigenin-induced Noxa activation in EGFRm tumor cells.

### Apigenin and ABT-263 inhibit tumor growth in vivo

**Fig. 8:**
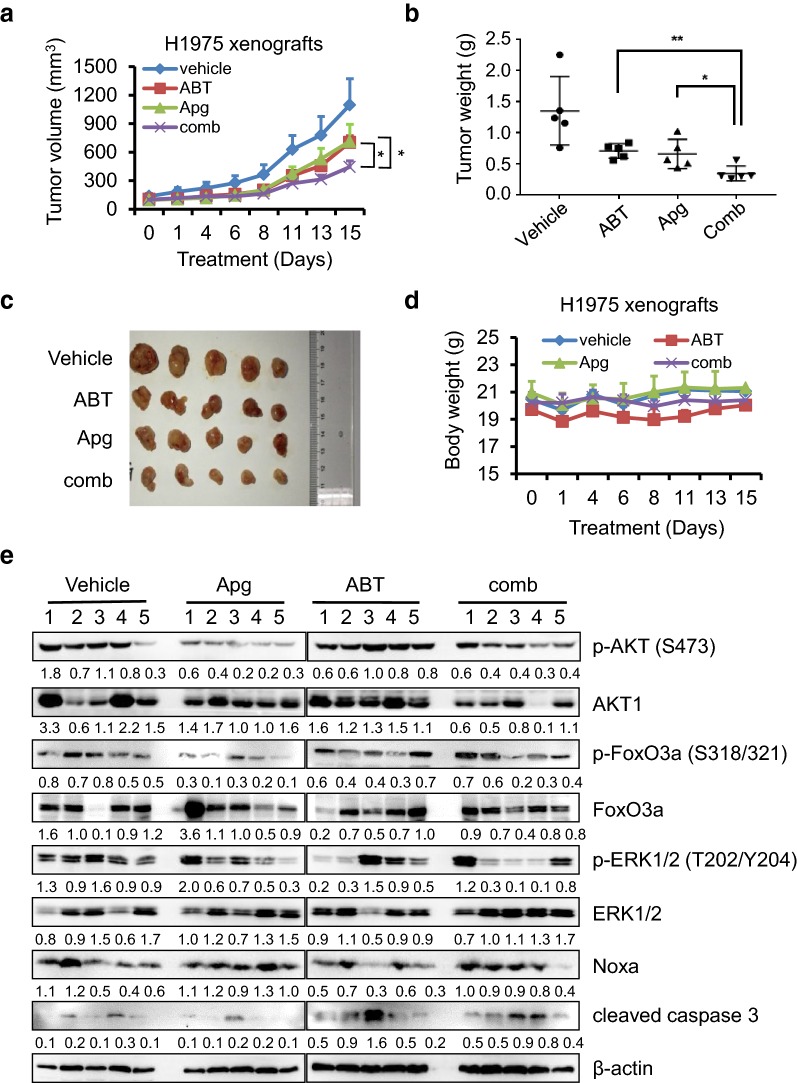
Apg and ABT-263 significantly suppress xenograft tumor growth in vivo. Nude mice bearing H1975 xenografts were grouped and treated with vehicle, ABT-263 (100 mg/kg), Apg (25 mg/kg) or combination therapy daily by oral gavage for 15 days. The tumor volume (**a**) and body weight (**d**) were monitored every 2–3 days. The data represents mean ± SD (n = 5). *p < 0.05, and **p < 0.01. At the end of the experiment, tumors were harvested and weighed (**b**, **c**). **e** Total protein lysates were prepared, and the expression levels of p-AKT, AKT, p-ERK1/2, ERK1/2, p-FoxO3a, FoxO3a, cleaved caspase 3 and Noxa were examined by Western blotting. β-Actin was detected as an endogenous loading control. Densitometry measurements, normalized to β-actin, are indicated below the corresponding blot

To explore the antitumor effects of apigenin and ABT-263 in vivo, we constructed a nude mouse subcutaneous tumor model using H1975 cells. As shown in Fig. 8a, single drug treatment inhibited tumor growth by approximately 38% and 36%, respectively, and the cotreatment of apigenin and ABT-263 significantly inhibited tumor growth by approximately 68%. When the experiments were terminated, the tumors were harvested and weighed. As shown in Fig. 8b and c, the combination treatment of apigenin and ABT-263 significantly suppressed tumor growth compared with single-drug treatment, further indicating that the cotreatment of ABT-263 and apigenin showed significant antitumor efficacy in EGFRm tumors in vivo. To assess the toxicities mediated by the cotreatment in vivo, the mouse body weight was measured every 2–3 days, and results showed no significant difference among various groups (Fig. 8d), suggesting that the drugs had little toxic side effects on those mice. The tumor tissues were also examined by Western blotting. As shown in Fig. 8e, the inactivation of AKT and ERK and dephosphorylation of FoxO3a were observed in the groups treated with apigenin alone or the cotreatment of apigenin and ABT-263. Additionally, Noxa upregulation and caspase 3 cleavage were demonstrated, consistent with in vitro results from established EGFRm tumor cell lines.

## Discussion

**Fig. 9:**
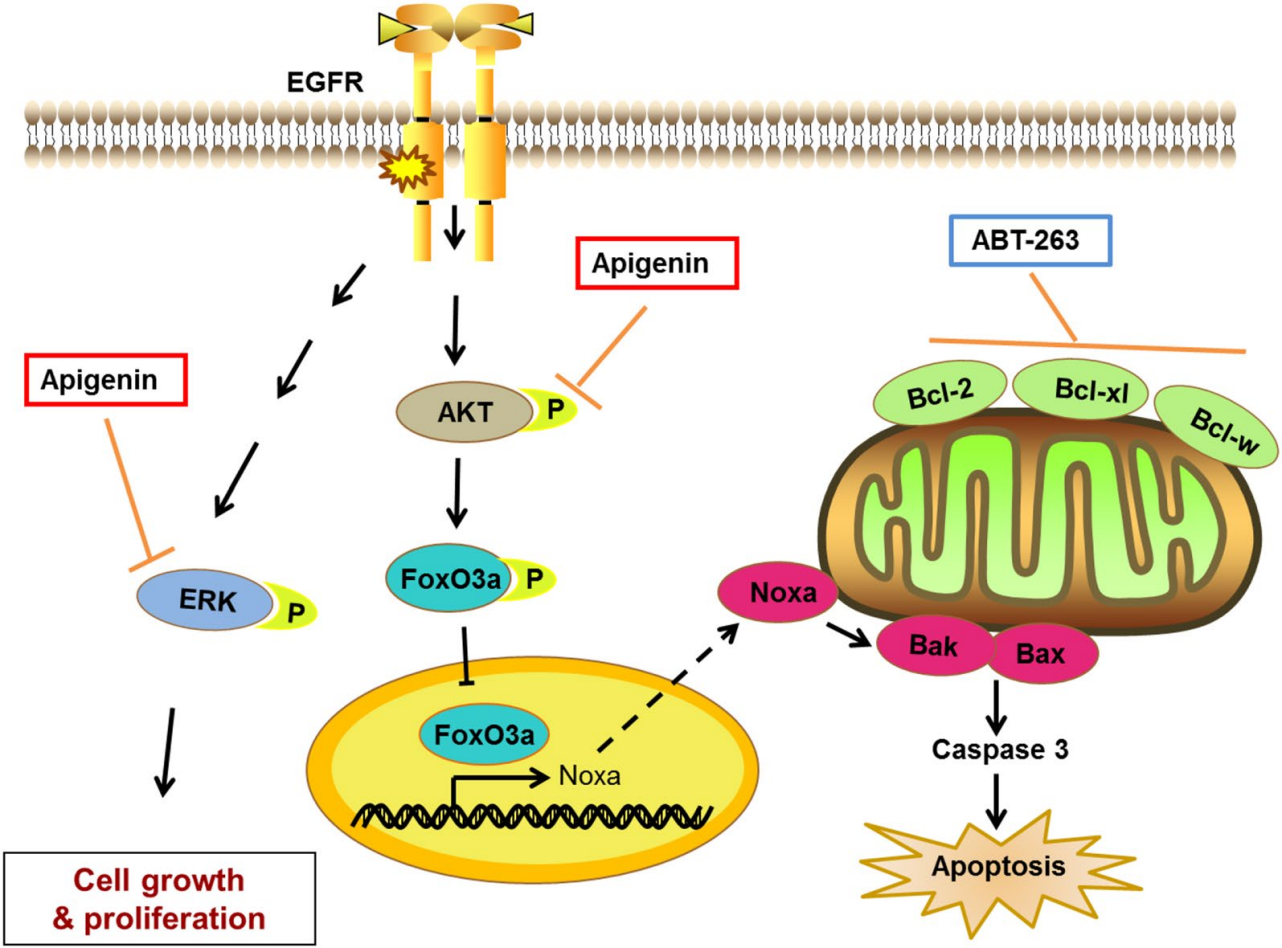
A model for synergistic interaction between apigenin and ABT-263 in cells with EGFR activating mutation. Apigenin upregulated the expression of Noxa in EGFRm tumor cells by targeting the AKT-FoxO3a pathway and inhibited ERK, thereby synergizing with ABT-263 to suppress tumor cell growth and proliferation in vitro and in vivo

Although the targeted therapy of EGFRm tumors by TKIs has achieved remarkable results in clinical applications in recent years, the acquired drug resistance to EGFR TKIs has been one of the urgent clinical challenges to be solved. In this study, we found that apigenin alone has only a mild inhibitory effect on EGFRm tumor cells (Fig. 1). By drug screening, we found that ABT-263 can significantly enhance the antitumor efficacy of apigenin in tumor cells harbouring activating EGFR mutations (Figs. 2, 3). Additionally, the cotreatment of apigenin and ABT-263 significantly inhibited the growth and proliferation of AZD9291-resistant H1975 cells (Fig. 4). Mechanistically, we discovered that apigenin can upregulate the expression of Noxa, thereby synergistically enhancing the effect of ABT-263 in EGFRm tumor cells (Fig. 5). Furthermore, we validated that apigenin enhanced the nuclear assembly of FoxO3a by the inactivation of AKT, thereby enhancing the expression of Noxa (Figs. 6, 7). Finally, the cotreatment of apigenin and ABT-263 was examined in vivo using a H1975 xenograft model. The coadministration of apigenin and ABT-263 significantly suppressed tumor growth compared with single-drug treatment (Figs. 8, 9).

Apigenin has a wide range of antitumor effects in various cancers. Considering that apigenin can inhibit the activation of AKT, ERK or STAT3, the major downstream signaling pathways of EGFR, we first analyzed the antitumor effects of apigenin on EGFRm tumor cells. Our data showed that, although apigenin showed significant dose-dependent inhibition of EGFRm tumors cells, the IC50 values are much higher than the peak plasma concentration (C_max_) of apigenin in vivo [27, 28]. Therefore, we carried out drug screening to explore the combination partner that could synergize with apigenin to enhance its antitumor effects. We then identified that ABT-263 can significantly enhance the cytotoxicity of apigenin in EGFRm tumor cells. ABT-263 is a Bcl-2 family protein inhibitor and one of the ingredients frequently evaluated in combination therapy studies for antitumor application [35]. Notably, our previous report showed that ABT-263 and apigenin could synergistically act against colorectal cancer [36]. According to statistical analysis of the COSMIC database, colorectal carcinoma cells contain high rates of the gene mutation of KRAS (34%), PIK3CA (14%) and BRAF (10%), the major downstream signaling molecules of EGFR. Those results also suggest that the synergistic antitumor effects of apigenin and ABT-263 on EGFRm tumors might occur through targeting the downstream signaling pathways of EGFR.

To date, various ADR mechanisms have been reported, with EGFR acquiring a second mutation leading to EGFR TKI resistance being an important cause [37]. For example, a biopsy of first-generation EGFR TKI-resistant patients revealed an EGFR T790M mutation in approximately 41–62% of cases [38, 39]. Additionally, different preclinical and clinical studies have shown that mutated tumors may cause resistance in several other ways, including small cell lung cancer (SCLC) transformation [39, 40], MET amplification [41, 42], HER2 amplification [42, 43], PIK3CA mutations [43], NRAS mutations and KRAS gain [44], BRAF mutations [45], and NF-κB activation [46]. In a recent study, Chabon et al. exploited CAPP-SeqctDNA analysis to study resistance mechanisms and revealed a largely underestimated heterogeneity of mechanisms of acquired resistance to 3rd generation EGFR TKIs, with the co-existence of multiple mechanisms in the same patient at a higher frequency than previously reported [47]. Notably, we found no new nonsense mutation sites in the H1975-AR cells. Although the mechanism by which H1975-AR cells resist AZD9291 treatment remains unclear here, it is obvious that the combination of apigenin and ABT-263 has strong antitumor effects on both EGFR TKI-sensitive and -resistant cells, further demonstrating that the combination of apigenin and ABT-263 might be a promising strategy to treat EGFRm tumors and overcome EGFR TKI resistance.

The function of FoxO3a is regulated by a complex network of processes, including post-translational modifications (PTMs) and protein–protein interactions. Additionally, its subcellular localization is important for its activities and functions [48]. The phosphorylation of FoxO3a leads to its translocation from the nucleus to the cytoplasm, where it associates with 14-3-3 protein, and this binding prevents its reentry into the nucleus [49, 50]. AKT is one of the key players to phosphorylate FoxO3a and regulate its subcellular localization and transcriptional activities [31, 32]. In this study, we found that apigenin could enhance FoxO3a nuclear accumulation by the inactivation of AKT, showing similar effects of the AKT inhibitor MK-2206. Additionally, the overexpression of ca-AKT1 significantly rescued EGFRm cells from the cell apoptosis triggered by the cotreatment of apigenin and ABT-263, further indicating that the AKT-FoxO3a signaling pathway plays a key role in the antitumor activities induced by the cotreatment of apigenin and ABT-263. Interestingly, as reported previously, ERK can interact with and phosphorylate FoxO3a, which increases FoxO3a-MDM2 interaction and enhances FoxO3a degradation, leading to tumorigenesis [51], suggesting that ERK promotes tumorigenesis by inhibiting FoxO3a. Unfortunately, we didn’t find that FoxO3a expression was regulated by ERK inactivation in our study (data not shown).

Additionally, we found that, among all the Bcl-2 family members, only Noxa was upregulated by apigenin alone or in combination. Noxa is a BH3-only member that has been proposed to play a key role in the control of apoptosis and initiation of apoptotic pathways [52]. As reported previously, various drugs could upregulate Noxa expression and contribute to the BH3 mimetics of ABT-263 or ABT-737 sensitivity [53, 54]. The knockdown of Noxa greatly decreased cell cytotoxicity induced by the cotreatment of apigenin and ABT-263 in EGFRm tumor cells, further indicating that Noxa plays a key role in the combination-caused antitumor efficacy. Furthermore, our data indicated that Noxa upregulation by apigenin was associated with the dephosphorylation of FoxO3a, confirmed by FoxO3a knockdown by shRNA inhibiting the apigenin-induced Noxa upregulation. Additionally, the AKT inhibitor of MK-2206 inhibited the phosphorylation of FoxO3a and upregulated Noxa expression in a time-dependent manner, further suggesting that Noxa expression is closely related to the AKT-FoxO3a signaling pathway.

## Conclusion

Together, the results of the present study show that apigenin could upregulate Noxa expression by inhibiting the AKT-FoxO3a signaling pathway, thereby synergizing with ABT-263 to exert antitumor effects in EGFRm tumor cells. Our studies provided a strong rationale for the clinical application of the combination treatment of apigenin and BH3 mimetic in the treatment of EGFRm tumors.

## Materials and methods

### Cell culture

EGFRm containing cell lines (H1975, HCC827, H1650, H3255, SK-MEL-28) and normal EGFR containing cell lines (HCT116, A549, Skov3, Panc10.05) were obtained from the American Type Culture Collection (ATCC). PASMC cells were kindly provided by Dr. Mingxiong Guo (Wuhan University, China). And BaF3 cells were kindly provided by Dr. Xianjun Fang (Virginia Commonwealth University). Cells lines were cultured in RPMI-1640 or McCoy’s 5A media (Hyclone, Logan, UT, USA) supplemented with 10% FBS (Biological Industries, Shanghai, China) and 1% penicillin/streptomycin (Solarbio, Beijing, China) and cultured in a humidified incubator at 37 °C containing 5% CO_2_. All cell lines were frozen at early passages and used for less than a month in continuous culture.

### Reagents

Apigenin, ABT-263, PD0325901, MK-2206 and AZD9291 were purchased from Selleck (Selleck Chemicals, Houston, USA). Fetal bovine serum (FBS) was obtained from BI (Biological Industries, Shanghai, China). Antibodies of cleaved PARP (#5625), cleaved caspase3 (#9661), Mcl-1 (#39224), Bcl-xL (#2764), Survivin (#2808), Bim (#2933), Noxa (#14766), STAT3 (#12640), p-STAT3 (Y705) (#9145), p-EGFR (Y1068) (#2234), EGFR (#4267), p-ERK1/2 (T202/Y204) (#4370), ERK1/2 (#4695), p-AKT (S473) (#4060) and p-FoxO3a (#9465) were obtained from Cell Signaling Technologies (Beverly, MA, USA). The antibody of Bcl-2 (#AB40639) was from Aboci (Aboci, MD, USA). Bax (#23931-1-AP), β-actin (#60008-1-lg), β-tubulin (#10094-1-AP) and FoxO3a (#10849-1-AP) was obtained from Proteintech (Wuhan, Hubei, China). AKT1 (#sc-5298) was purchased from Santa Cruz (Santa Cruz Biotechnology, Santa Cruz, California). 4′,6-Diamidino-2-phenylindole (DAPI) were obtained from Solarbio (Beijing, China).

### Colony formation assays

Single cells in 24-well plates (200 cells per well) treated with the indicated agents (or DMSO) and incubated at 37 °C in 5% humidified CO_2_. Media were changed every 3–4 days. After 10–14 days, colonies were washed with PBS twice and fixed with methanol for 15 min, then stained with 0.1% crystal violet. The colony formation ability was presented as percentage compared with DMSO treatment group. Three independent experiments were performed in triplicate.

### Cell viability and apoptosis assays

Cell viability was determined using the MTT assay, and cell proliferation was determined using the Operetta High Content Imaging System. For the MTT assay, 1 × 10^4^ cells were seeded in 96-well plates and were treated with the indicated drugs for 48 or 96 h. Next, 10 μL per well of MTT (12 mM in PBS) was added. After incubation at 37 °C for 2–4 h, 100 μL of SDS-HCl solution was added, the mixtures were further incubated at 37 °C for 2-4 h, and then the absorbance at 570 nm was recorded using a microplate reader (ELX808, BioTek, Winooski, VT, USA).

For cell apoptosis assay, H1975 and HCC827 were seeded in 6 wells plates with 1.5 × 10^5^ cells per well. Following drug treatment, both floating and adherent cells were collected and stained with Annexin V-FITC/propidium iodide (PI) according to the manufacturer’s instruction (#FXP018, 4A Bioteck, Beijing, China). The stained cells were analyzed by flow cytometry (ACEA NovoCyte, USA). The extent of apoptosis was quantified as a percentage of annexin V-FITC positive cells.

### Western blotting analysis

Following appropriate treatment, cells were harvested and lysed and subsequent protein was quantified with the BCA protein assay (Solarbio, Beijing, China) and western blotting was performed as described previously [55]. Densitometric analysis for protein quantification was performed using ImageJ Software. Cleaved PARP and cleaved caspase 3 are normalized to β-actin or β-tubulin, respectively. And others are normalized to β-actin or β-tubulin and then compared to vehicle-treated control, indicated below the corresponding blot. The data showed in the manuscript are representative of at least three independent experiments.

### Plasmids construct and lentivirus packing

Plasmids of pCDH-EGFR-wt, -G719S, -T790M, -L858R and -T790M/L858R were generated from the pBabe EGFR (-wt, -G719S, -T790M, -L858R and -T790M/L858R; Addgene, Watertown, Massachusetts) and cloned into pCDH-CMV-MCS-EF1-Puro plasmid (System Biosciences) respectively using standard molecular cloning techniques. Plasmid of pCDH-EGFR-C797S was prepared from pCDH-EGFR-wt using overlapping PCR [56]. All the constructs were verified by sequencing.

The pGreenPuro shRNA plasmid was obtained from System Biosciences. The shRNA lentivirus vectors were generated by cloning target-specific oligonucleotides into the lentiviral vector according to the manufacturer’s instructions. The target sequences of the Noxa and FoxO3a shRNA template oligonucleotide used were as follows: sh-Noxa: 5′-GTAATTATTGACACATTTCTT-3′; sh-FoxO3a: 5′-GTCACTGCATAGTCGATTCAT-3′. The details of sh-ctrl and sh-Bax were described previously [36]. The viruses were produced by cotransfection of 293TN cells with lentiviral vector, packing and envelope plasmids using Lipofectamine 2000 (Life Technology) as previously described [36]. The virus-containing supernatants were harvested 2–3 days post transfection and used for infection of cells or stored in aliquots at − 80 °C.

### Real-time PCR

Total RNA was extracted using a total RNA Simple Kit according to the manufacturer’s instructions (TIANGEN, Beijing, China). And the cDNA was prepared from total RNA using PrimeScript™ RT Master Mix (#RR036A, TaKaRa, Shiga, Japan). Real-time PCR was performed on Bio-rad iCycler 4 Real-Time PCR Detection System (Bio-Rad, Hercules, CA) using SYBR Green I. Primers used were as follows: Noxa: 5′-GCTGGAAGTCGAGTGTGCTA-3′ (forward) and 5′-GGAGTCCCCTCAATGCAAGTT-3′ (reverse). GAPDH: 5′-GGGAAGGTGAAGGTCGGA-3′ (forward) and 5′-GCAGCCCTGGTGACCAG-3′ (reverse). The Noxa expression was presented as fold changes normalized to GAPDH mRNA and compared with control group.

### Immunofluorescence staining

H1975 cells fixed with methanol were incubated with FoxO3a (1:100 dilution; Proteintech, 10849-1-AP) antibody and then with goat anti-rabbit secondary antibody conjugated with Alexa Fluor 488. After washing with PBS, H1975 cells were stained with DAPI (1 µg/mL) and analyzed under a super resolution confocal microscope (Dmi8, Leica, Wetzlar, Germany).

### Xenograft study

All animal protocols were approved by the Institutional Animal Care and Use Committee of Shaanxi Normal University. Experimental studies were carried out using 6- to 8-week-old female BALB/c nude mice with an average body weight of 20 g obtained from Beijing Vital River Laboratory Animal Technology. H1975 cells (5 × 10^6^) were implanted subcutaneously at the flank region. Tumor growth was monitored by periodic visual inspection at the site of implantation, and the dimensions of the xenografts were measured every 2 to 3 days. Tumor volume was calculated using the following formula: volume (mm^3^) = 0.5 × longest tumor diameter × (shortest tumor diameter)^2^. The mice were randomized into four groups (n = 5 per group) and were treated with vehicle, apigenin, ABT-263 alone or the combination of ABT-263 and apigenin. Apigenin was dosed at 25 mg/kg and ABT-263 at 100 mg/kg. Both agents were formulated in 10% DMSO (V/V), 0.5% methyl cellulose (V/V), and 89.5% ddH_2_O (V/V) and administered by oral gavage daily. For the combination treatment, the agents were formulated at a higher concentration so that the total volume of the two agents together was equivalent to the volume of each agent alone. At the endpoint, the mice were euthanized, and the tumors were harvested for weight and Western blotting analysis.

### Statistical analysis

All the experimental data are presented as mean ± SD for at least three independent experiments performed in triplicate. The coefficient of drug interaction (CDI) was calculated as described previously [57]. There was considered a significant synergistic effect of a two-drug combination for CDI < 0.7. The statistical significance of the differences between two groups was determined using Student’s unpaired two-tailed *t* test. *p *< 0.05 was considered statistically significant.

## Additional file

**Additional file 1.** Additional figures.

## Data Availability

All data generated or analyzed during this study are included in this published article and its additional file.

## Abbreviations

EGFR: epidermal growth factor receptor
EGFRm: activating EGFR mutation
apg: apigenin
NSCLC: non-small cell lung cancer
STAT3: signal transducer and activator of transcription 3
RTKs: receptor tyrosine kinases
TKIs: receptor tyrosine kinase inhibitors
FoxO3: forkhead box O3
MAPK: mitogen-activated protein kinase
ERK: extracellular signal-regulated kinase
PI3K: phosphoinositide 3-kinase
DMSO: dimethyl sulfoxide
Mcl-1: myeloid cell leukemia-1
PD1: programmed cell death 1
PD-L1: programmed death ligand 1
