# Supplementary material for: BH3 mimetic ABT-263 enhances the anticancer effects of apigenin in tumor cells with activating EGFR mutation
Source: Cell Biosci. 2019 Jul 23;9:60. doi: 10.1186/s13578-019-0322-y (PMC6651933; doi:10.1186/s13578-019-0322-y)
Supplement: Supplementary file 1 — Additional file 1. Additional figures. [file 13578_2019_322_MOESM1_ESM.pdf]

Additional file 1: Figure S1

a

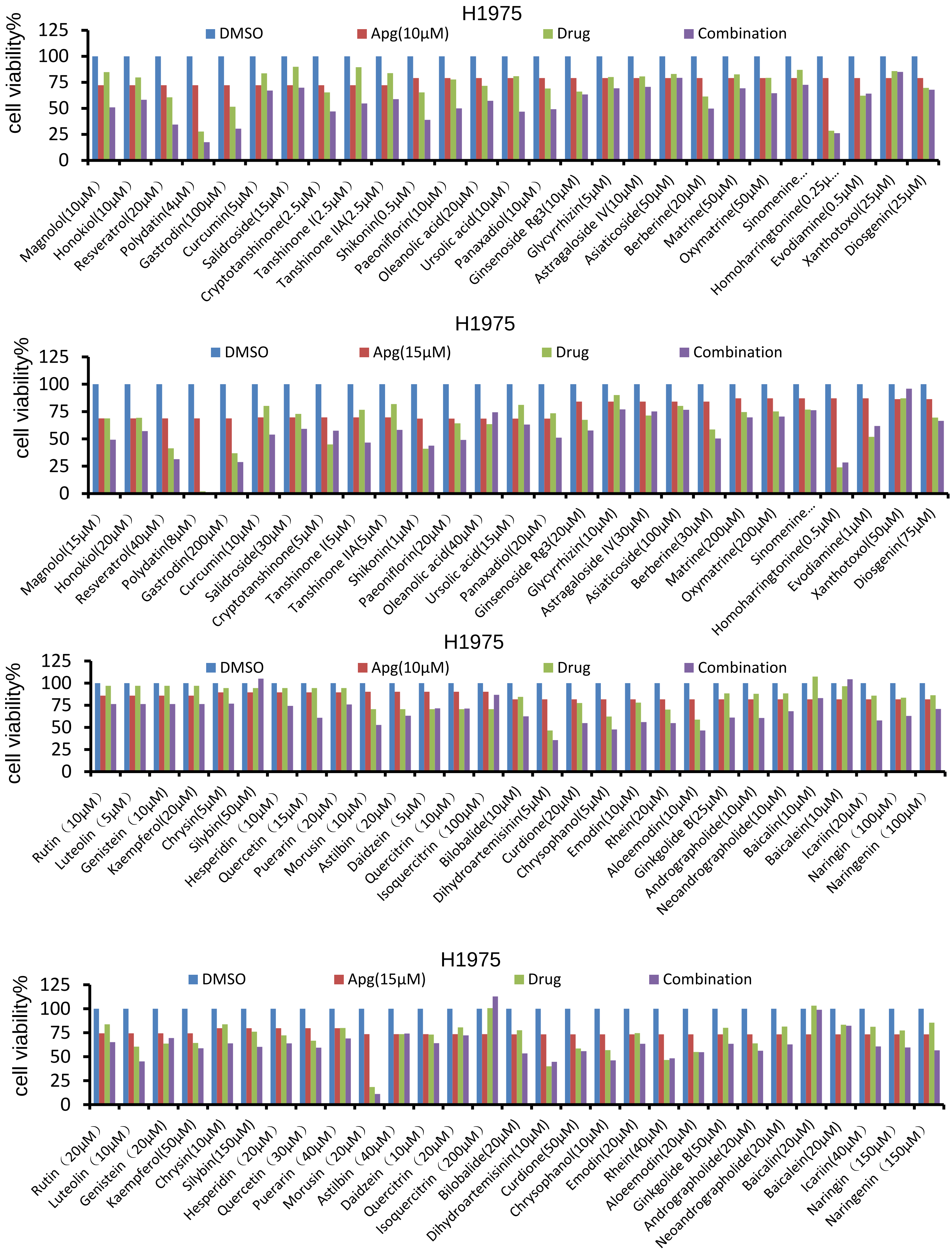

b

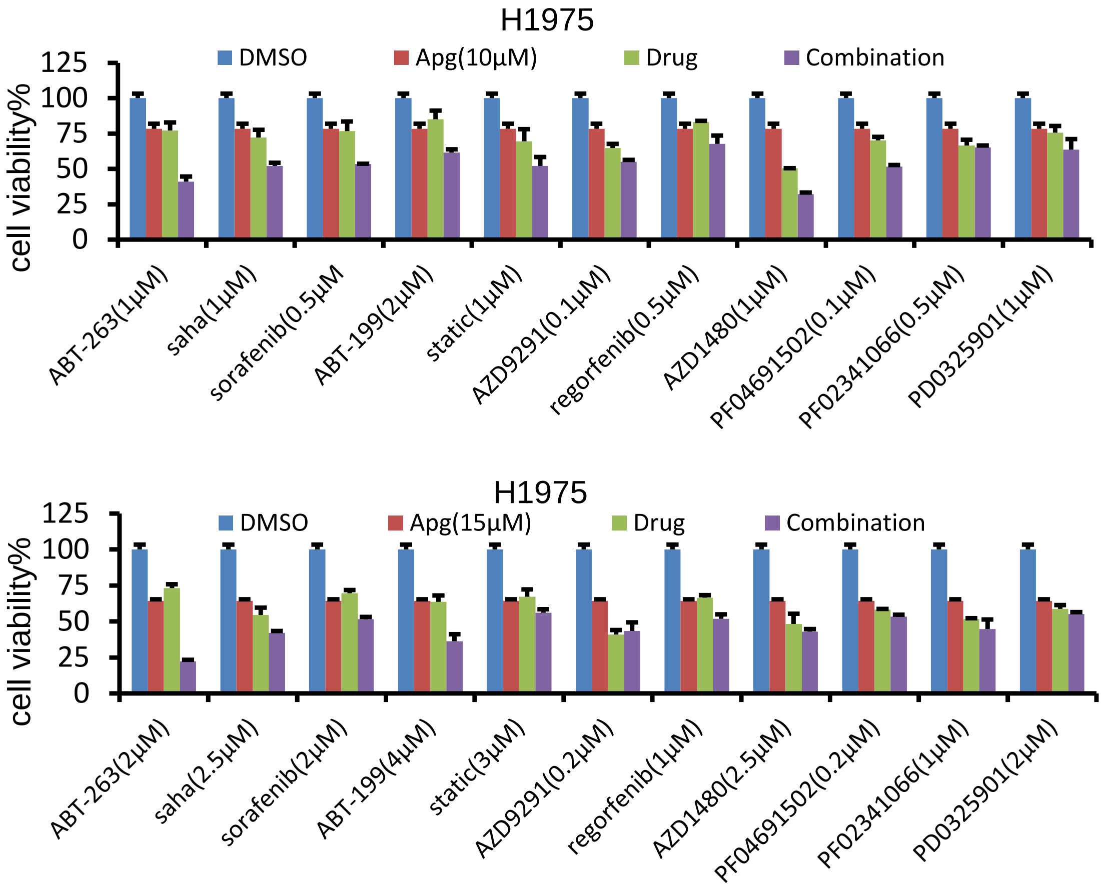

c

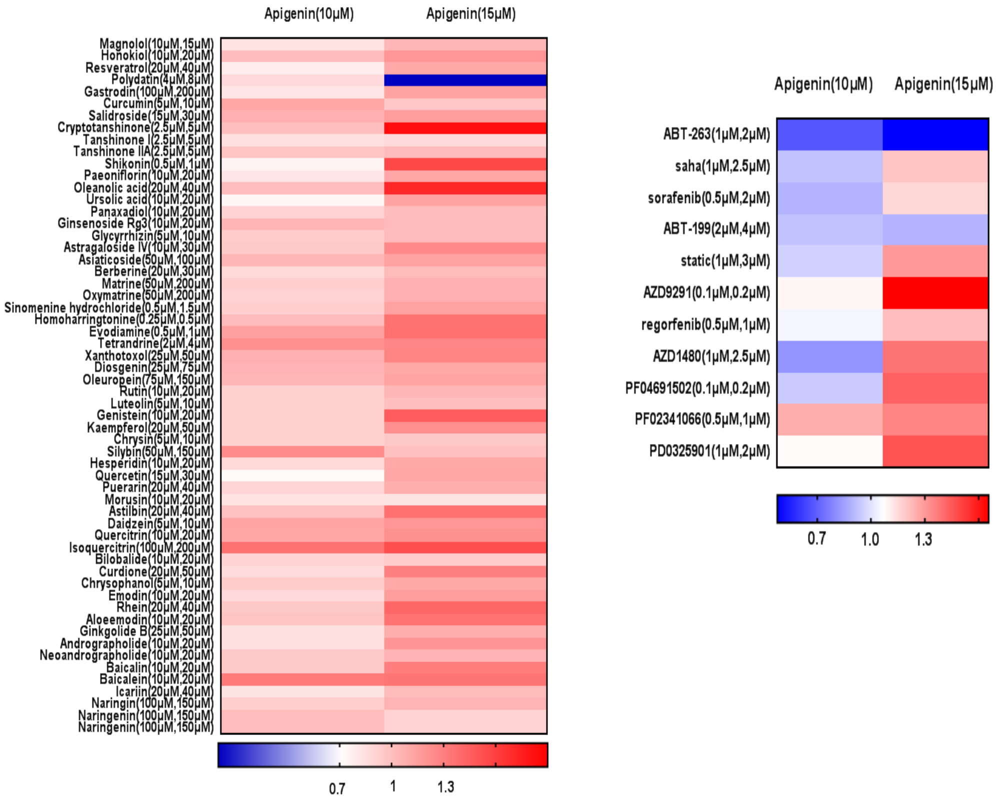

Additional file 1: Figure S1. ABT-263 enhances Apigenin-induced anti-tumor activities through drug screening. (a, b) H1975 cells were treated with Apg (10 μM, 15 μM), compounds, alone or the combination as indicated for 2 days. Cell viability rates were determined by MTT assay. (c) Heatmap showing the Coefficient of Drug Interaction (CDI) for the combination of Apigenin (10μM, 15μM) with other agents in H1975 cells. A CDI value of < 1, =1 or >1 indicates a synergistic, additive effect or antagonistic effect, respectively.

Additional file 1: Figure S2

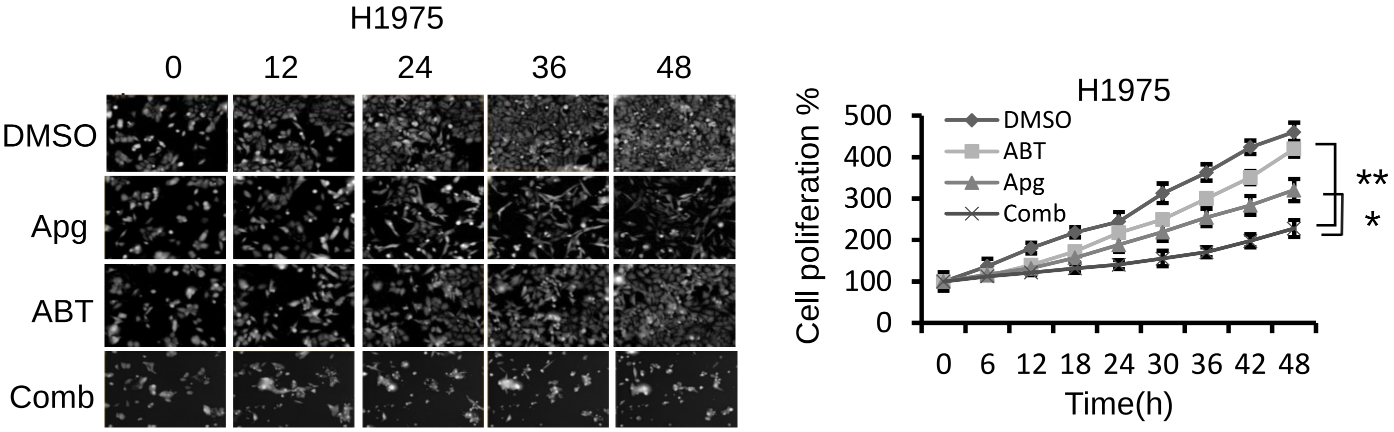

Additional file 1: Figure S2. Coadministration of ABT-263 and apigenin significantly suppressed tumor cell proliferation. H1975 cells were seeded in 24-well plates and were treated with Apg (15 µM) and ABT (2 µM), alone or in combination for 2 days. Representative pictures at indicated time points (0, 12, 24, 36, 48h) are shown by the Operetta High Content Imaging System and quantified by Harmony High Content Imaging and Analysis software. The data represents mean ± SD (n=5) . * indicates $p < 0.05$, and ** indicates $p < 0.01$ vs single compound treatment group.

Additional file 1: Figure S3

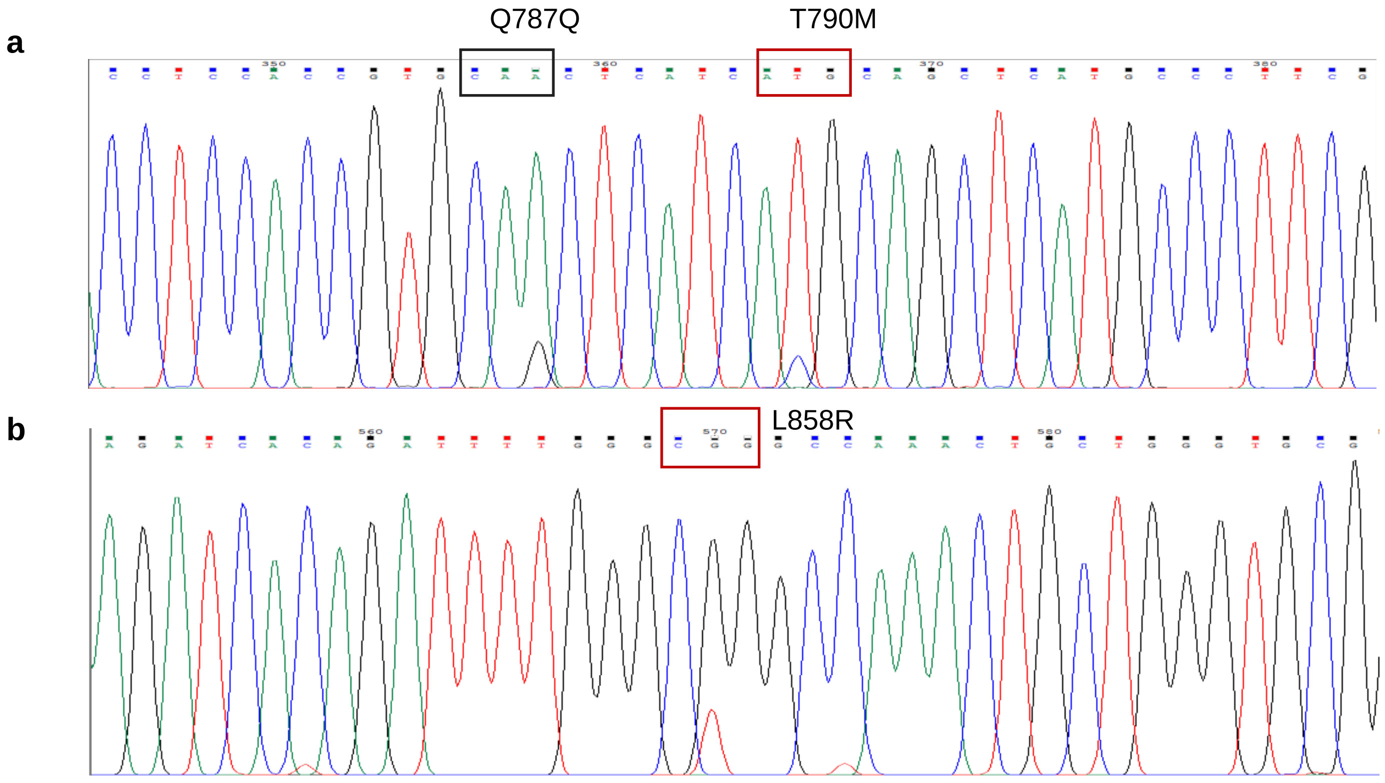

Additional file 1: Figure S3. Identification of AZD9291-resistant H1975 cells. (a, b) EGFR sequencing analysis showed that there is a same-sense mutation at codon 787 in addition to the two known mutation sites of T790M and L858R in H1975-resistant cells.

Additional file 1: Figure S4

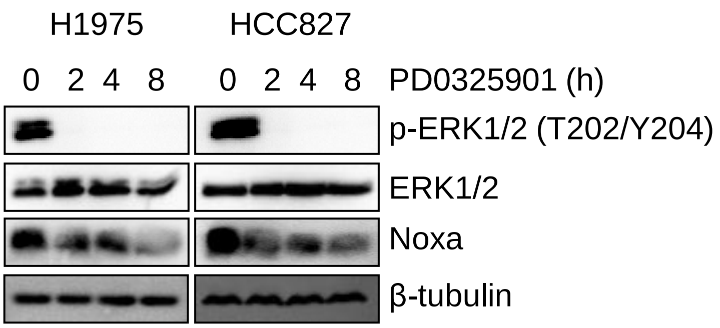

Additional file 1: Figure S4. ERK inhibition by PD0325901 did not upregulate Noxa expression. H1975 and HCC827 cells were treated with PD0325901 (2 μM) for up to 8 h. Cells were harvested, and the expression levels of ERK and Noxa were examined by Western blotting.
